# Sex Pheromone of Cocoa Pod Borer, *Conopomorpha cramerella*: Field Activity Evaluation of Pheromone Formulations in an Indonesia Plantation

**DOI:** 10.3390/insects13080663

**Published:** 2022-07-22

**Authors:** Jerome Niogret, Arni Ekayanti, Aijun Zhang

**Affiliations:** 1 Mars Wrigley, Nguma-bada Campus, James Cook University, Smithfield, QLD 4878, Australia; 2 Centre for Tropical Environmental & Sustainability Science, Nguma-bada Campus, James Cook University, Smithfield, QLD 4878, Australia; 3 Mars Cocoa Research Centre, Mars Wrigley, Tarengge 92971, Sulawesi Selatan, Indonesia; arni.ekayanti@effem.com; 4 Invasive Insect Biocontrol and Behavior Laboratory, United States Department of Agriculture, Agricultural Research Service, Beltsville, MD 20705, USA; aijun.zhang@usda.gov

**Keywords:** cocoa pod borer, field evaluation, pheromone lure formulation, dose-response, longevity

## Abstract

**Simple Summary:**

We have investigated the efficacy of six different pheromone lures for the cocoa pod borer in Indonesia. The lures provided by the USDA captured more males than the lures manufactured by Alpha Scents when loaded with a 0.1 mg pheromone blend. We increased the lure longevity to 7 months by increasing the Alpha Scents pheromone load to 1 mg. The long-life pheromone lure may be particularly useful in monitoring large-scale cocoa farms and developing new mitigation technologies that would necessitate high longevity powerful attractant.

**Abstract:**

The previously identified female sex pheromone of cocoa pod borer (CPB), *Conopomorpha cramerella* (Snellen) (Lepidoptera: Gracillariidae), was re-evaluated for male attraction using six different pheromone formulations in Indonesian cocoa plantations. In a dose-response experiment, the 0.1 mg pheromone load was significantly more attractive to male CPB than the lower doses tested. Additionally, during the first four-week trapping period, USDA (Beltsville, MD, USA) lures containing 0.1 mg of synthetic pheromone blend exhibited significantly better attraction than the commercial lure obtained from Alpha Scents, Inc. (Canby, OR, USA) with the same pheromone load (0.1 mg). Although the 1.0 mg lure did not show any higher attraction than the 0.1 mg lure during the first month, it was significantly attractive for CPB males with the same weekly average capture efficacy for the whole twenty-seven weeks in field conditions in 2018. A long-life pheromone lure can be particularly useful in monitoring large-scale cocoa farms.

## 1. Introduction

Mention of trade names or commercial products in this article is solely for the purpose of providing specific information and does not imply recommendation or endorsement by the US. Department of Agriculture.

The cocoa pod borer (CPB), *Conopomorpha cramerella* (Snellen) (Lepidoptera: Gracillariidae), has been reported as the most serious insect pest of cocoa in Southeast Asia, and losses can be well over 50% of the crop [1]. The sex pheromone of *C. cramerella* was identified in 1986 [1,2,3], and mass trapping using pheromone was reportedly effective in reducing CPB infestation in large-scale pilot studies (>200 ha) in East Malaysia [4]. However, the use of pheromones against *C. cramerella* was halted in the early 1990s, partly due to economic reasons and poor-quality control of the commercial pheromone preparations that later failed in field trials. Additional testing found that the synthetic pheromone was less effective in field tests conducted in West Malaysia, indicating possible strain differences affecting lure efficacy [5]. Zhang et al. [6] found no regional differences in field tests conducted in Malaysia and Indonesia but cited lack of commercial quantities and problems with quality control of synthetic pheromone components as issues that may limit lure efficacy.

Besides its economic importance, the cocoa pod borer’s behavior and ecology are under-documented [7,8].

Various control methodologies have been tested to control the CPB infestation with relatively limited success. The use of parasitoids or entomopathogens did not provide enough efficacy or was not economically viable [9,10,11]. Enhancing the black ant population to increase predation and disturbance showed conflicting results [12,13,14]. Covering the cocoa pods with plastic or biodegradable sleeves successfully prevent CPB oviposition [15], but many farmers consider this method too labor-intensive [16,17]. Cultural practices such as pruning, sanitation, complete and synchronous harvesting of mature pods can temporarily reduce pest populations until the CPB population gets reintroduced from neighboring farms or alternative hosts [18].

Consequently, CPB control mainly depends on the heavy use of pesticides despite the uncertain cost/efficiency ratio. Topical insecticides often have a limited impact on the larval development occurring within the cocoa pod protection [4,19,20]. More sustainable control methods for CPB should be developed due to increasing concerns about pesticide residues in cocoa beans. Meanwhile, pesticide applications directly related to the crop’s phenology and the CPB population density should be prioritized over regular and systematic applications without knowledge of the pest abundance in the field. Qualitative and quantitative assessments of yield with an understanding of how it is affected by pests and diseases is a *sine qua non* condition for the development of efficient crop management and IPM strategies [21,22]. The cocoa industry needs to know the effects of particular pests and diseases on crop yields in quantitative terms, so the appropriate measures can be applied when necessary to avoid the predicted losses [23,24,25]. This action threshold is determined by detecting and estimating the pest densities with accurate monitoring tools, and the observed pest-related damages obtained from scouting external and internal symptoms on the cocoa pods.

This study is part of a collaboration started in 2004 with the United States Department of Agriculture that helped restart the work on pheromones to develop an efficient CPB management. The objectives were to re-evaluate the attractive activity of the sex pheromone of *C. cramerella* in Indonesia and Malaysia to determine if the commercial formulations could be used as an accurate and efficient monitoring system. This article describes the field trapping studies that examined and compared the efficacy, variability, and longevity of two pheromone formulations and several pheromone doses in cocoa fields in Indonesia.

## 2. Materials and Methods

### 2.1. Lure Preparation

The USDA pheromone lures were synthesized and formulated at the IIBBL, Maryland, USA. The polyethylene vials (26 × 8 × 1.5 mm thick, Just Plastic Ltd., Norwich, UK) were loaded with 10, 30, 50, and 100 μg of CPB synthetic sex pheromone blend [(*E,Z,Z*- and *E,E,Z*-4,6,10-hexadecatrienyl acetates and corresponding alcohols with ratio of ~ 40:55:4:6)] and an equal weight of 2,6-di-tert-butyl-4-methylphenol (BHT) as an antioxidant [6]. The impurity of non-target geometric isomers was 47%, and the lids of the vials were closed during the entire experimental period [26]. The Alpha Scents formulations were developed by Alpha Scents Inc. (Canby, OR, USA) using the same pheromone components and similar ratio as described [1], but with different polyethylene vials (LDPE microcentrifuge vial, 30 × 5 × 1.5 mm thick, Thermo Fisher Scientific, Waltham, MA, USA). Both 100 μg and 1 mg synthetic pheromone blend doses were used in those experiments.

### 2.2. Field Location

Field trapping experiments were conducted in Luwu, South Sulawesi, Indonesia, at a local cocoa farm locally named Insitu (−2.545520; 120.792481). The field was a 1 ha cocoa farm composed of 15-year-old cocoa trees separated by 3 m from each other and irregularly shaded by banana, coconut, and Durian trees. The farm was surrounded by other cocoa farms with similar shaded trees.

### 2.3. Field Experiments

Experiment #1 consisted of two consecutive similar field tests conducted in February (Experiment 1, Test 1) and October 2019 (Experiment 1, Test 2) in Insitu to assess the efficacy of various pheromone doses (10, 30, 50 and 100 µg) in the USDA pheromone lure compared to unbaited traps (control). The experiments were carried out until no significant differences in trap catch could be observed between the pheromone lures and the unbaited control traps. The USDA pheromone lures were hung from wires 3 cm above the center of the sticky liners of white plastic delta traps (28 × 20 × 15 cm, ISCA Technologies, Riverside, CA, USA). The same delta traps and liners were used in the rest of the experiments. The two field tests were set in February and October 2019 and contained five replicates of each of the five treatments, each replicate consisting of a row of traps with treatments randomized, and 12 m between traps within a replicate and between replicates. Traps were hung approximately 1 m above the tree canopy from a PVC pole attached to the tree’s trunk. The number of captures was checked, and traps were rotated weekly sequentially to minimize the potential positional effect.

Experiment #2 corresponded to the efficacy comparison of the 100 μg pheromone lures from two different sources, the USDA and Alpha Scents, and compared to unbaited traps (control). The experiment was set up in Insitu and contained 10 replicates of each of the three treatments. Each of the 5 rows of traps included 2 randomized replicates per treatment, and 12 m between traps within and between replicates. Traps were hung approximately 1 m above the tree canopy from a PVC pole attached to the tree trunk and separated by 12 m from each other, within and between rows. Both tests were lasted for 4-weeks, the numbers of captures were checked, and traps were rotated weekly to minimize the potential positional effect.

Experiment #3 compared efficacies between Alpha Scents lures loaded with two pheromone blend quantities: 100 μg and 1 mg, to the USDA’s 100 μg pheromone lure used as a positive control, and to unbaited traps (negative control). This experiment contained 10 replicates of each of the four treatments, each replicate consisting of a row of traps with treatments randomized, and 12 m between traps within and between replicates. The numbers of captures were checked, and traps were rotated weekly to minimize the potential positional effect. The experiment continued until there was no significant difference with the unbaited traps.

### 2.4. Statistical Analysis

The male CPB capture was transformed by log(x + 0.5) to hold the Normality assumption [27]. The USDA pheromone lure efficacy was compared with unbaited blank-control traps using Student *t*-tests. The effect of the lure type on the moth captures was analyzed using a generalized linear model (glm) with time used as a continuous predictor. One Way-ANOVA followed by Fisher LSD Post-hoc tests (Statistica 12; StatSoft, Tulsa, OK, USA) was used to test the significance within time periods. Results were given as mean ± SE.

## 3. Results

**Figure 1:**
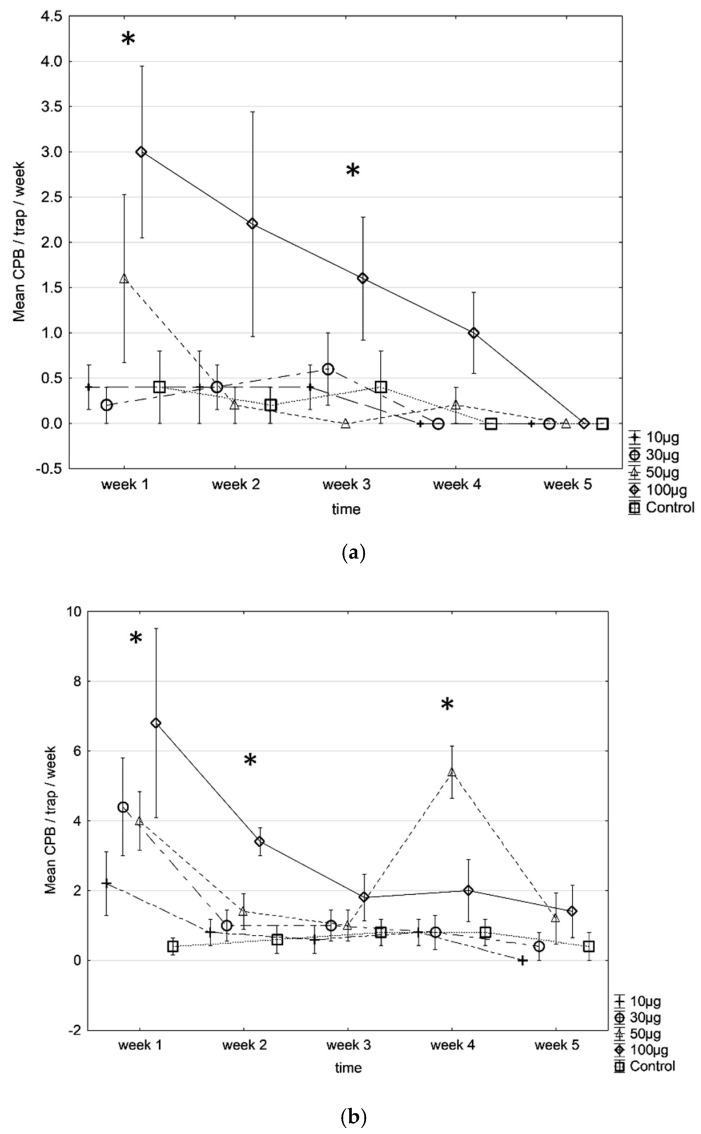
Mean captures of CPB (±SE) in traps baited with 10, 30, 50, and 100 µg USDA pheromone lures against untreated blank-control traps for 4-weeks. Both comparative field trials were done at the Insitu farm during two different periods: (**a**) February 2019 (*Experiment*#1, Test 1) and (**b**) October 2019 (*Experiment*#1, Test 2). Each treatment was repeated five times in both field experiments. * *p* < 0.05.

Tests 1 & 2 of experiment #1, conducted during low and high CPB population density seasons respectively, showed that the traps baited with 100 µg dose captured the most males. During the low-density season, the only dose attracting significantly more males was 100 µg, while there were no differences between the two lower doses and the unbaited traps (glm, F(4, 119) = 8.81, *p* < 0.001) (Figure 1a). When the pest population was higher (Figure 1b), both traps baited with 50 and 100 µg lures captured the highest number of insects, significantly more than the traps baited with 10 µg dose and blank control traps (glm, F(4, 119) = 7.92, *p* < 0.001). The traps baited with 10 µg dose did not capture more males than the unbaited traps over the 4-weeks.

The efficacy of the pheromone lures loaded with various doses was tested for five weeks, as the significance with the unbaited blank control traps disappeared at week 5. During the low population density season (Figure 1a), the trap baited with 100 µg pheromone lure was the only one significantly capturing more CPB than the unbaited blank control trap after the first week of the experiment (F(4, 20) = 3.50, *p* = 0.025), then the average of captures decreased regularly. At week 5, there was no difference between any of the pheromone lures and the unbaited blank control trap.

The same pattern was observed in the season with a higher pest population (Figure 1b), the same pattern was observed. All pheromone-baited traps captured CPB in the first week, with the traps baited with 100 µg lure capturing the most males. At week 3, no differences in trap catches were observed for all traps. Surprisingly, at week 4, all the traps baited with 50 µg lures captured significantly more CPB than any other treatments (F(4, 20) = 10.55, *p* < 0.001). While a contamination cannot be excluded while rotating the traps, no satisfactory explanation was found. This difference disappeared in week 5.

**Figure 2:**
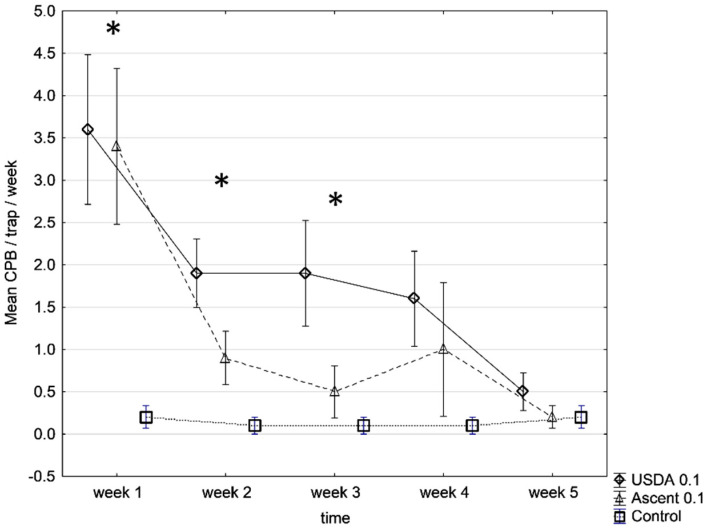
Mean captures of CPB (±SE) in traps baited with 100 µg USDA and 100 µg Alpha Scents pheromone lures against untreated traps. The field trial was conducted in December 2017 at the Insitu farm (*Experiment*#2). * *p* < 0.05.

In experiment #2, the trap capture showed the same pattern as the previous tests, with a significant effect of the lure type on the trap capture during the whole experimental period (glm, F(2, 146) = 29.99, *p* < 0.001). The traps baited with USDA pheromone lures captured the highest number of males in the first week, before progressively losing their attractiveness and averaging at 2.3 ± 0.4 males/trap/week after 4 weeks. The traps baited with 100 µg Alpha Scents pheromone lures were catching moths at a similar rate as the traps baited with USDA lures in the first week (t = 0.397; df = 18; *p* = 0.696) and significantly more than unbaited blank-control traps (t = 5.514, df = 18; *p* < 0.001), while losing their attractive potential after three weeks (t = 1.224, df = 18; *p* = 0.237). Over the 4-week experiment period, the traps baited with 100 µg Alpha Scents lures captured a 1.5 ± 0.5 males/trap/week (Figure 2).

**Figure 3:**
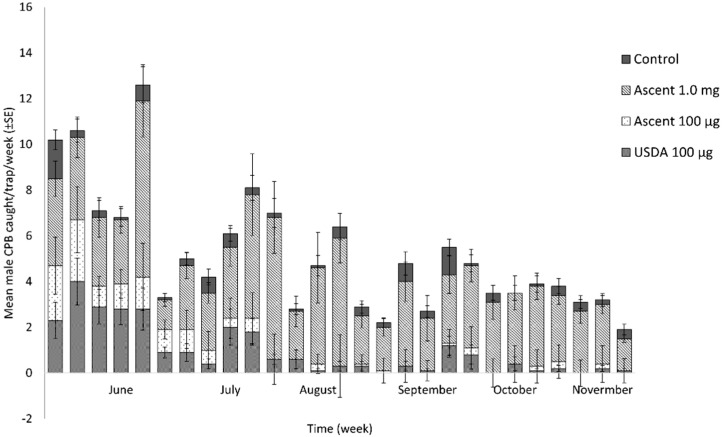
The flight activity and mean captures of CPB (±SE) in Delta traps baited with 100 µg and 1 mg Alpha Scents pheromone blend and 100 µg USDA blend in Insitu farm. The test was conducted from May 2018 to November 2018 (*Experiment*#3), and traps were rotated weekly.

In experiment #3, the traps baited with the Alpha Scents’ and USDA’s 100 µg pheromone lures started capturing 2.3 ± 0.8 and 2.4 ± 1.2 males/trap the first week respectively, and were significantly more attractive than the blank control traps for a period of 4 and 6 weeks, respectively (t = 2.583, df = 18; *p* = 0.019, and t = 3.327, df = 18; *p* = 0.004, respectively). The traps baited with Scents’ and USDA’s 100 µg pheromone lures captured an average of 1.8 ± 0.8 and 2.6 ± 0.7 males/trap/week over their attractive periods for USDA, respectively (Figure 3).

The traps baited with 1 mg Alpha Scents pheromone lure started with a similar capture rate to the traps baited with lower 100 µg lures (F2, 27) = 0.84; *p* = 0.441), but with a higher average capture rate (4.44 ± 1.00 males/trap/week) over the 5 weeks. The 1 mg pheromone lures were significantly more attractive than the lower pheromone doses and the blank control up to 28 weeks with a 3.2 ± 0.8 males/trap/week on average for lure lifespan (glm, (F(3, 1115) = 196.20, *p* < 0.001) (Figure 3).

## 4. Discussion

Our experiments demonstrated that the 100 μg pheromone lures were systematically more attractive than the lower dosages tested. This capture effectiveness difference was especially true when the CPB adult population density was at its lowest season (February), and larger pheromone quantities had to be available to attract the scarcely distributed males at a longer distance. During the higher population density season (October in Sulawesi), the 50 μg pheromone lures were enough to attract the same number of males as the 100 μg pheromone lures. The adult population of CPB is considered relatively low all around the year, with Beevor et al. [2] estimating a high density at only 200 pairs/ha in Sabah, Malaysia. A scouting effort was made on a smallholder’s farm in South Sulawesi from March to June 2022 to estimate the number of CPB adults. It was found that the estimated population grew from 70 to 470 pairs/ha during this period [28]. The few CPB males attracted by caged calling females in experiments conducted in Indonesia and Malaysia also confirmed the low CPB population density Niogret [29] captured 8.26 males/trap/week in Sulawesi, Indonesia, in March-April 2018, comparable to the 6.8 males/trap/week reported in Sabah Malaysia [2]. Three previous evaluations [6,26,29] of the pheromone lure efficacy for CPB used the same pheromone source, quantity and vial formulations as we have used in this study. Vanhole et al. [30] demonstrated that the USDA pheromone blends with 47% impurity used in [26] and in the present study had the same efficacy in capturing males as the 5% impurity of non-target geometric isomers used in [6]. While the trapping density differed, the comparison shows consistency. In 2008, Zhang et al. [6] published an extensive field evaluation of the CPB pheromone lures in Indonesia and Malaysia. Overall, the study showed an average capture rate of 4.9 males/trap/week in 4 locations in Sulawesi, Indonesia, using ≈12 m inter-trap distance in 2006; an average of 2.53 males/trap/week in two locations in Peninsula Malaysia in 2005; and an average of 3.45 males/trap/week in four locations in Sabah, MCB. Later on, Vanhole et al. [30] reported CPB captures between 2.01 ± 0.26 (±SE) and 3.61 ± 0.77 males/trap/week using a 11.25 traps/ha density, in Sabah, Malaysia, using the traps baited with two levels of pheromone purities during 12 February–16 May 2013; and 1.75 ± 0.28 capture/trap/week and 2.62 ± 0.38 in another experiment from 13 August–4 March, 2013–14. In 2019, Vanhole et al. [26] average captured fluctuated between 0.8 and 6.9 males/trap/week in a standard management plot in Pahang Province of peninsular Malaysia in two main cocoa harvest seasons, from 14 November–6 May, 2014–15, and 5.6 males/trap/week between 20 October and 3 May, 2015–16, using 2 traps/ha replaced every 10 weeks. Those numbers are relatively low but generally consistent over the years. Our current study was done in Sulawesi and showed similar captures to the previous studies in Sulawesi, averaging between 1.8 and 3.4 males/trap/week between 2017 and 2019, with a similar trap density as [6]. The highest capture ever reported in the literature was from Zhang et al. [6] in the Rambong Sialand Estate, Sumatra, Indonesia, in 2006 over 5 weeks, where 120 males/trap/week were recorded without any changes in the lure attractiveness in the first four weeks, and an average 54 males/trap/week in two locations of Teck Guan Estates, Sabah, Malaysia during a year-long evaluation in 2006. The lures used in the 2006 field evaluation were the same as those used in [30]. Therefore, a high population density seemed to be the primary explanation for such capture rates reported in [6].

The pheromone lure longevity followed the same pattern in all our field experiments. There was a significant decline in insect trap capture after the first-week lure deployment and a progressive capture reduction for the three remaining weeks. These capture rate reductions occurred in both low and high population density seasons. Our 4–5-week lure longevity using the 100 μg pheromone lures was similar to the 1–2-month longevity reported by Zhang et al. [6] and Vanhole et al. [30] in both Sabah and Peninsular, Malaysia.

Our dose-response study revealed an increased efficacy from 10 to 100 μg, reaching a maximum capture at 100 μg. In a previous study, the same capture efficacy between the 100 μg and 1 mg pheromone lures was reported, suggesting that the higher doses of the sex pheromone blend in the vial formulation did not increase the male trap catch [6]. Based on the pheromone components desorption obtained in the lab laboratory, Zhang et al. [6] estimated that the longevity of a lure with a 1 mg pheromone load could last around 4–5 months. This estimation was close to what we empirically observed in Sulawesi, Indonesia. Our longevity experiment was done with an Alpha Scents vial formulation, slightly different from the USDA lure used in the authors’ estimation. Our 1 mg sex pheromone formulation was significantly attractive for 7 months. While a trade-off exists between the financial costs of loading the lures with more pheromone versus the increased longevity, improving the lure longevity would bring important insight into using pheromone lures as potential mitigation measures.

Vanhole et al. [30] demonstrated that the USDA pheromone blends with 47% impurity of non-target geometric isomers they used had the same efficacy in capturing male CPB as the 5% impurity lures obtained from the UK and used in [6]. Our study confirmed the CPB attractive activity using the same ‘impure’ USDA pheromone blend. This discovery may be ground-breaking as a pheromone blend synthesized by a lower-cost synthetic pathway, and more impurity of non-target geometric isomers could still provide the same effectiveness and offer new perspectives of using CPB pheromone lures in the mitigation system against the cocoa pod borer. Reducing the cost of pheromone production will enable growers to use CPB monitoring tools with higher pheromone loading. While a ten-fold increase in the pheromone load did not improve our CPB trap capture rate in the first month, our study showed a significant extension of the pheromone lure longevity up to 7 months. Such extended longevity offers new mitigation perspectives like mass-trapping and mating disruption technologies. Beevor et al. [4] found a significant decline in male captures and cocoa pod damages in mass-trapping trials, while Mumford and Beevor [31] speculated that preventing at least 5% loss would make a mass-trapping cost-effective. Similarly, Alias et al. [32] demonstrated promising results in reducing the female mating ratios using CPB pheromone lures in a mating disruption trial. Vanhole et al. [26] also tested the CPB pheromone lure in an attract-and-kill system alongside cypermethrin. They successfully demonstrated that it could reduce the number of infested cacao pods compared to standard insecticide management. A potential combination of pheromonal compounds with female-attractive plant kairomones would integrate mating disruption and female removal technologies, as demonstrated for *Cydia pomonella* (Tortricidae) [33,34].

## 5. Conclusions

All the encouraging results of using pheromone-based mitigation systems for CPB would benefit from the extensive longevity found in this study using the 1 mg lure, which is usually the limiting factor for mass-trapping, mating disruption, and attract-and-kill control methodologies. The steady CPB male capture for up to 7 months, coupled with the cost-effective synthetic pathway described by Vanhole et al. [30], may bring those control methods economically viable for environmentally friendly cocoa production.

## Data Availability

The data supporting this study’s findings are available from the corresponding author, Jerome Niogret, upon reasonable request.
